# Application of multivariate joint modeling of longitudinal biomarkers and time-to-event data to a rare kidney stone cohort

**DOI:** 10.1017/cts.2022.465

**Published:** 2022-09-26

**Authors:** Lisa E. Vaughan, John C. Lieske, Dawn S. Milliner, Phillip J. Schulte

**Affiliations:** 1 Department of Quantitative Health Sciences, Mayo Clinic, Rochester, MN, USA; 2 Division of Nephrology and Hypertension, Mayo Clinic, Rochester, MN, USA; 3 Department of Laboratory Medicine and Pathology, Mayo Clinic, Rochester, MN, USA

**Keywords:** Joint models, survival analysis, biomarkers, kidney failure, primary hyperoxaluria

## Abstract

**Background:**

Time-dependent Cox proportional hazards regression is a popular statistical method used in kidney disease research to evaluate associations between biomarkers collected serially over time with progression to kidney failure. Typically, biomarkers of interest are considered time-dependent covariates being updated at each new measurement using last observation carried forward (LOCF). Recently, joint modeling has emerged as a flexible alternative for multivariate longitudinal and time-to-event data. This study describes and demonstrates multivariate joint modeling using as an example the association of serial biomarkers (plasma oxalate [POX] and urinary oxalate [UOX]) and kidney function among patients with primary hyperoxaluria in the Rare Kidney Stone Consortium Registry.

**Methods:**

Time-to-kidney failure was regressed on serially measured biomarkers in two ways: time-dependent LOCF Cox proportional hazards regression and multivariate joint models.

**Results:**

In time-dependent LOCF Cox regression, higher POX was associated with increased risk of kidney failure (HR = 2.20 per doubling, 95% CI = [1.38-3.51], p < 0.001) whereas UOX was not (HR = 1.08 per doubling, [0.66–1.77], p = 0.77). In multivariate joint models, estimates suggest higher UOX may be associated with lower risk of kidney failure (HR = 0.42 per doubling [0.15–1.04], p = 0.066), though not statistically significant, since impaired urinary excretion of oxalate may reflect worsening kidney function.

**Conclusions:**

Multivariate joint modeling is more flexible than LOCF and may better reflect biological plausibility since biomarkers are not steady-state values between measurements. While LOCF is preferred to naïve methods not accounting for changes in biomarkers over time, results may not accurately reflect flexible relationships that can be captured with multivariate joint modeling.

## Introduction

Cox proportional hazards regression is a popular statistical method used in kidney disease research to evaluate biomarkers associated with progression to kidney failure [1]. For studies in which biomarkers are obtained in a longitudinal manner (serially over time), methods such as time-dependent multivariable Cox models are often used to model time-to-kidney failure, with the longitudinal biomarkers of interest considered as time-dependent covariates updated at each new set of measurements. This method allows for modeling of unevenly spaced measures during follow-up and across patients, and the process utilizes the last observation carried forward (LOCF) method, thus assuming that the biomarker measures remain at a constant level until the next measurement [2]. These time-dependent Cox model approaches are superior to time-invariant analyses that instead model combinations of the longitudinal biomarker as fixed at baseline, leading to incorrect and biased conclusions [3].

While the time-dependent multivariable Cox model is superior to naïve time-invariant analyses, the LOCF assumption, or similar assumptions that result in biomarkers only being updated at measured times, may be adequate for evaluation of some short-term mechanistic associations, but may not accurately reflect the true functional relationships of many interrelated time-dependent biomarkers and outcomes, particularly when the biomarker measurements are sparsely recorded over an extended period of time [3]. Moreover, as biomarker measurements throughout follow-up are commonly ascertained based on physician judgement or in response to patient symptoms rather than fixed by design, it is important that the factor(s) influencing biomarker measurement be accounted for in the model [4].

Recently, multivariate joint models have received increased interest due to their flexibility for handling longitudinal and time-to-event data [5,6]. By modeling the joint distribution of biomarkers obtained over time and the survival event process, both the functional form as well as the visit process mechanism can be explicitly specified [7], allowing comparison of longitudinal biomarker measures on the event of interest. In the multivariate joint model, this is expanded, allowing comparison of multiple longitudinal biomarkers on the time-to-event. This model capability can be particularly useful when the biomarker measures are interdependent and not regularly measured at the same time points.

The goal of this study was to evaluate plasma oxalate (POX) and urine oxalate (UOX) as risk factors for developing kidney failure independent of estimated glomerular filtration rate (eGFR) among patients with primary hyperoxaluria (PH) type 1 (PH1). PH1 is a rare genetic metabolic disorder characterized by hepatic overproduction of oxalate due to mutations in the *AGXT* gene that encodes alanine glyoxylate transferase. This excess oxalate cannot be further metabolized by humans and instead must be eliminated by the kidneys. Thus, UOX reflects hepatic oxalate production, and since PH1 patients excrete high concentrations of oxalate in the urine, they are at high risk for calcium oxalate kidney stones and progressive oxalate nephropathy chronic kidney disease (CKD) (lower eGFR). Patients with PH1 often experience kidney failure [8]. Prior literature suggests that the magnitude of UOX is associated with CKD risk when renal function remains relatively good (CKD stages 1–3a; eGFR ≥ 45 ml/min/1.73 m^2^), but this biomarker may not be useful in latter CKD stages 3b–5 (eGFR < 45 ml/min/1.73 m^2^) due to reduced renal oxalate excretion [9]. POX is influenced by both the amount of hepatic oxalate production and the ability of the kidneys to clear it, and increases markedly as eGFR falls below 45 ml/min/1.73 m^2^ [9]. Thus, POX may be more informative in PH1 patients in the more severe CKD stages 3b–5 due to its causal role in systemic oxalate deposition (oxalosis), whereas the utility of monitoring POX in less severe CKD stages remains to be established [9,10]. In clinical practice, the laboratory measures POX, UOX, and eGFR are not recorded on a pre-set schedule but are instead measured based on clinical indication during the patient’s disease course, often driven by patient-specific factors and events. Further, POX, UOX, and eGFR are often not measured at the same time, making it difficult to do comparative assessments across measures. Thus, we apply multivariate joint modeling to assess the association of UOX and POX with progression to kidney failure. Results are compared to the time-dependent Cox regression approach. In this paper, we describe our data and provide an overview of analytic strategies including multivariate joint modeling, and compare results describing the estimated association between POX and UOX biomarkers and time-to-kidney failure in this cohort.

## Methods

### Data

We studied PH1 patients enrolled in the Rare Kidney Stone Consortium (RKSC) PH Registry [10,11]. Our cohort includes patients 2 years or older with PH1 who were free of kidney failure at the time of PH1 diagnosis and had at least one eGFR measure available between 1 year prior to PH1 diagnosis and prior to kidney failure (Supplemental Figure S1). Kidney failure was defined as the first occurrence of transplant, initiation of dialysis, or eGFR < 15 ml/min/1.73 m^2^. Patients were censored at last follow-up or death; death without a kidney failure event is rare in this population (two events, 1.2%). GFR was estimated from serum creatinine using Pottel’s full age spectrum equation, which allows for estimation of the GFR across all ages [12].

This study was approved by the institutional review board at Mayo Clinic, Rochester.

### Analytical Strategies

Two approaches for analysis of time-to-event (kidney failure) data regressed on multiple serially collected biomarkers (POX, UOX, eGFR) were compared: time-dependent multivariable Cox proportional hazards models and multivariate joint models for longitudinal and survival data.

#### Time-dependent multivariable Cox regression

**Fig. 1.**
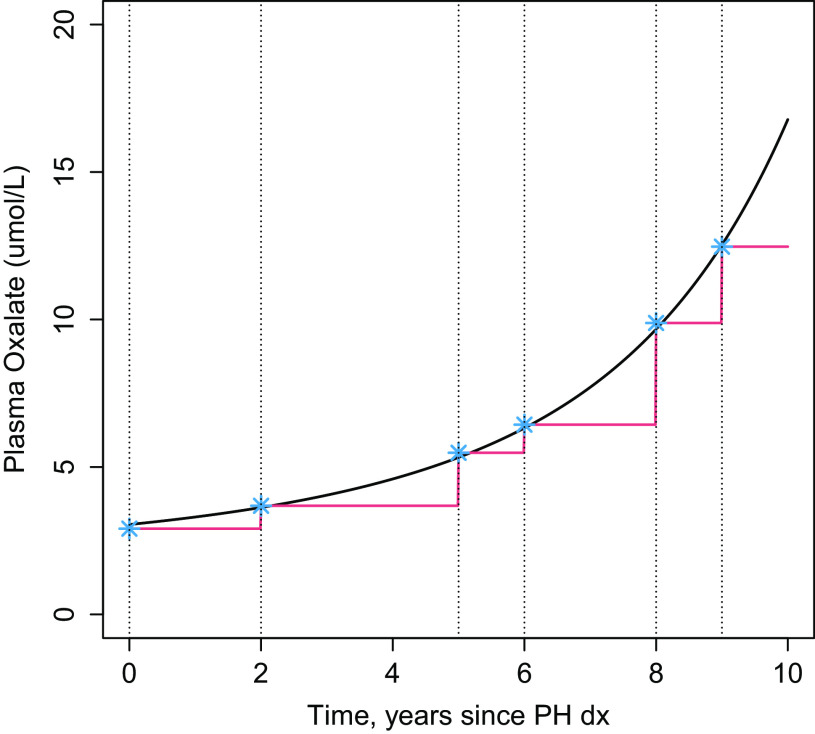
Serially measured plasma oxalate (POX) for a hypothetical primary hyperoxaluria (PH) patient. Points plotted in blue denote observed POX measures at baseline, 2, 5, 6, 8 and 9 years after PH diagnosis. The red line indicates the time-dependent biomarker value used in the model for time-to-kidney failure using the LOCF approach. The black line indicates the subject-specific predicted values of POX using the joint modeling approach. During times when POX is changing rapidly (years 6 through 10 in the hypothetical patient) or measured infrequently, the LOCF approach poorly approximates the true value.

We first assessed the relationship between serial biomarker data and progression to kidney failure using multivariable Cox regression, with time-dependent covariates for POX, UOX, and eGFR. One of the strengths of this modeling approach as compared to naïve methods is the ability to incorporate covariates that change over time by using “last value carried forward,” such that the last known value of each covariate is used forward in time until a new value is measured [2]. Investigators can also use other time-dependent variables if supported by the study hypothesis, such as time-dependent cumulative average. However, the underlying assumption of this stepped function process may not be optimal for estimating an association between a time-dependent covariate and kidney failure, for example when the biomarker may not remain at a steady state value between observations (Fig. 1). Moreover, imputation using LOCF methods may not yield valid inferences when the probability of observing or not observing a longitudinal biomarker at a particular time depends on unobserved longitudinal responses, particularly when more than one time-dependent covariate is being measured [7].

#### Multivariate joint modeling using a Bayesian approach

We also performed multivariate joint modeling of longitudinal and time-to-event data. In brief, a joint model is two or more linked regression submodels [13]. We fit regression submodels for each serially measured biomarker (POX, UOX, and eGFR) and a submodel for the time-to-kidney failure outcome. They are linked through subject-specific random effects present in both the biomarker(s) and time-to-kidney failure submodels [13]. See the Supplemental Methods section for further details of how these equations were derived.

We illustrate in Fig. 1 a hypothetical patient with POX measured serially, with actual observed values plotted as the blue points at baseline, 2, 5, 6, 8, and 9 years after baseline. In the LOCF approach illustrated by the red line, the time-dependent biomarker value used in the model for time-to-kidney failure has jumps whenever the biomarker value is updated for the patient but is otherwise in a steady state between observations. However, the true biomarker process is likely smooth, and the black line may represent subject-specific predicted values from the longitudinal submodel at every timepoint. In the joint model, those subject-specific predicted values (black line) may be the time-dependent predictor for kidney failure, updated continuously over time.

Joint models with subject-specific predicted values allow more flexibility for inclusion of subjects with missing biomarker values at baseline, and/or sparse follow-up biomarker assessments. While power and precision will improve with greater frequency of biomarker observation, subjects may be included with as few as one observation of the biomarker. Joint modeling has been extended to handle multivariate longitudinal data, which traditionally has been computationally intensive, hence limiting its applicability. Whereas *multivariable* references a model with many predictors or covariates, a *multivariate* model is the analysis of multiple outcomes (either different outcomes or repeated measures) in a single fitted model. A model may be neither, one, or both multivariable and multivariate. A Bayesian approach enables fitting these multivariate joint models with the JMbayes package in R [14].

### Statistical Methods

For time-dependent Cox models using LOCF, univariable Cox models were separately fit for each biomarker measure, using years since PH1 diagnosis as the time scale. For multivariable models including more than one biomarker, follow-up began at the first instance a patient had measurements available for all biomarkers; if measurements were taken at different time points, then the previous biomarker measurement was carried forward in time (LOCF) until the other biomarkers(s) were first observed. That is, data were left truncated and subjects without observation of each biomarker prior to kidney failure were excluded from the model.

For joint models, longitudinal sub-models for each biomarker were fit along with a survival sub-model to assess the risk for a kidney failure event. Longitudinal submodels for each biomarker used linear mixed effects models with log-transformed POX, UOX, and eGFR to satisfy assumptions of normally distributed residuals. Random intercepts were used to account for the correlation of repeated observations from the same individual. Random slopes were considered but did not improve model fit. Covariates in the longitudinal models included age, sex, and time (in years) since PH1 diagnosis. The optimal functional forms for time in each model were assessed using natural cubic splines. The best functional form identified for time since PH1 diagnosis modeled for eGFR and POX was a natural cubic spline with 2 degrees of freedom (internal knot at the 50th percentile of the distribution), and for UOX was a natural cubic spline with 5 degrees of freedom (internal knots at the 20%, 40%, 60%, and 80% percentiles). Model fit was assessed using Deviance Information Criterion (DIC) values.

For the joint models, a survival submodel was fit for the outcome of time-to-kidney failure from PH1 diagnosis. The time-dependent subject-specific predicted mean biomarker values (POX, UOX, eGFR) were included as predictors. That is, rather than use LOCF which assumes a constant value of the biomarker between measurements, in joint models at any given timepoint the predictor takes on the value of the subject’s estimated biomarker value as a function of age, sex, and years since PH1 diagnosis plus an estimate of the subject-specific intercept. The survival submodel also adjusted for age and sex, while using years since PH1 diagnosis as the time scale. In survival submodels of the joint model and in the time-dependent Cox regression, all biomarkers evaluated as predictors of kidney failure (POX, UOX, eGFR) were natural log-transformed; reported hazard ratios (HR) are interpreted as the increase in hazard of kidney failure per doubling of the lab value calculated using the formula 2^*β*^. Other flexible relationships for the association between subject-specific longitudinal biomarker estimates and hazard for kidney failure are available using software, but these two (original scale and log-transformation) provide a natural interpretation and fit our data well. The proportional hazards assumption was checked for variables in survival models by plotting Schoenfeld residuals over time; no violations were suggested. Correlations of random intercepts – that is, the correlation of subject-specific random effects from different longitudinal submodels – are estimated in the multivariate joint model. We also examined these associations after restricting to follow-up occurring after a patient entered CKD stage 3a (eGFR < 60 ml/min/1.73 m^2^) as a sensitivity analysis.

In the main analysis, we conducted a complete case analysis under the assumption of baseline biomarkers missing completely at random, assuming site availability of oxalate testing rather than patient characteristics is the reason for missing data. However, we also conducted an analysis using multiple imputation assuming those without observation of a biomarker were missing at random, possibly related to other observed data. POX, UOX, and eGFR at PH1 diagnosis were imputed in 20 imputed datasets when data were missing; LOCF time-dependent Cox models and multivariate joint models were run on each imputed dataset and results were combined across imputations to reflect uncertainty due to missing data.

R code implementing the main analysis is provided in the **Supplemental Materials**. P-values were considered statistically significant at the two-sided 0.05 alpha level. All analyses were performed using R version 3.6.1 or higher (R Foundation for Statistical Computing, Vienna, Austria) and SAS 9.4 (SAS Institute Inc., Cary, NC).

## Results

Baseline characteristics of 166 patients meeting inclusion criteria are reported in Table 1. During a mean (SD) follow-up of 16.3 (1.4) years, 60 (36.2%) patients developed kidney failure. Only two patients (1.2%) died without a kidney failure event. There were 1285 eGFR measures throughout follow-up prior to kidney failure; median [IQR] number of eGFR measures per patient was 5 [2, 11]. Among the 111 patients with a POX measure during follow-up before kidney failure, 39 kidney failure events and a total of 555 POX values were recorded; median [IQR] number of POX measures per patient was 2 [1, 7]. Among the 150 patients with a UOX value recorded throughout follow-up prior to kidney failure, 55 kidney failure events occurred and a total of 1091 UOX values were recorded; median [IQR] number of UOX measures per patient was 5 [2, 11].

**Table 1.** Patient characteristics at primary hyperoxaluria (PH) diagnosis

Patient characteristic at baseline	N = 166
Age at PH diagnosis (years), mean (SD)	14.91 (15.03)
Sex, n (%)	
Male	87 (52.4%)
Female	79 (47.6%)
Race/Ethnicity, n (%)	
Asian	30 (18.1%)
Non-Hispanic White	110 (66.3%)
Hispanic (any Race)	8 (4.8%)
Other or Not Disclosed	18 (10.8%)

### Association between POX, UOX, eGFR, and Risk of Kidney Failure Using LOCF Time-Dependent Cox Regression

Results of analyses using time-dependent LOCF for biomarkers (Table 2) are expressed as per doubling of the biomarker. In univariable models, both POX and UOX were positively associated with risk of kidney failure. Specifically, a doubling of POX was associated with more than three times higher hazard for kidney failure (HR 3.27 [95% CI, 2.35–4.55]) and a doubling of UOX was associated with 74% higher hazard for kidney failure (HR 1.74 [95% CI, 1.30–2.34] per doubling). In multivariable models including all three biomarkers (POX, UOX, and eGFR), POX remained statistically significant with more than a two-fold increase in hazard of kidney failure per doubling of POX while UOX was no longer statistically significant after adjusting for POX and eGFR.

**Table 2.** Estimated hazard ratios per doubling of the biomarker (POX, UOX, eGFR [columns]) from univariable and multivariable last observation carried forward (LOCF) Cox models predicting risk of kidney failure, adjusted for age and sex. POX, UOX, and eGFR are time-dependent variables that update when new measurements are observed but assume a constant (LOCF) value between measurement. Rows represent models accounting for different biomarkers alone or in combination with one-another

Model	N	E	POX		UOX		eGFR	
			HR (95% CI)*	P	HR (95% CI)*	P	HR (95% CI)*	P
eGFR	166	60	–	**–**	**–**	**–**	**0.05 (0.03–0.09)**	<**0.001**
POX	111	39	**3.27 (2.35–4.55)**	<**0.001**	**–**	**–**	**–**	**–**
UOX	150	55	**–**	**–**	**1.74 (1.30–2.34)**	<**0.001**	**–**	**–**
eGFR + POX	111	39	**1.73 (1.18–2.54)**	**0.005**	**–**	**–**	**0.08 (0.03–0.22)**	<**0.001**
eGFR + UOX	150	55	**–**	**–**	1.25 (0.89–1.75)	0.20	**0.05 (0.02–0.09)**	<**0.001**
POX + UOX	106	38	**4.03 (2.71–5.99)**	<**0.001**	1.06 (0.69–1.61)	0.81	**–**	**–**
eGFR + POX + UOX	106	38	**2.20 (1.38–3.51)**	<**0.001**	1.08 (0.66–1.77)	0.77	**0.10 (0.04–0.26)**	<**0.001**

N = number of patients; E = number of events; eGFR = estimated glomerular filtration rate; UOX = urine oxalate; POX = plasma oxalate;

LOCF = last observation carried forward.

* HR can be interpreted as per doubling of the biomarker value.

Estimates in bold denote statistical significance at the 0.05 level.

Analyses using multiple imputation for missing data yielded similar conclusions (Supplemental Table S1). In the multivariable model including POX, UOX, and eGFR, our data suggested POX was associated with increased hazard for kidney failure (HR 1.49 [95% CI, 1.01–2.18] per doubling) whereas there was little evidence of an association between UOX and kidney failure, similar to the complete case analysis.

### Association between POX, UOX, eGFR, and Risk of Kidney Failure Using Joint Modeling

When analyzed individually using univariate joint modeling for biomarkers on the log scale (Table 3), POX and UOX were both positively associated with a greater risk of kidney failure (HR 2.78 [95% CI, 1.74–4.98] per doubling of POX and HR 1.74 [95% CI, 1.13–2.77] per doubling of UOX). However, in multivariate joint models, POX was no longer statistically significant after adjusting for UOX and eGFR (HR 1.91 [95% CI, 0.88–4.79]). The relationship between UOX and kidney failure was inverted compared to univariate analyses, although was not statistically significant after adjustment for both POX and eGFR (HR 0.42 [95% CI, 0.15–1.04]).

**Table 3.** Estimated hazard ratios per doubling of the biomarker (POX, UOX, eGFR [columns]) from univariate and multivariate joint models predicting risk of kidney failure, adjusted for age and sex. Rows represent models accounting for different biomarkers alone or in combination with one-another

Model	N	E	POX			UOX			eGFR		
			N labs	HR (95% CI)*	P	N labs	HR (95% CI)*	P	N labs	HR (95% CI)*	P
eGFR	166	60	–	**–**	**–**	**–**	**–**	**–**	1285	**0.10 (0.05–0.18)**	<**0.001**
POX	111	39	555	**2.78 (1.74–4.98)**	<**0.001**	**–**	**–**	**–**	**–**	**–**	**–**
UOX	150	55	–	**–**	**–**	1091	**1.74 (1.13–2.77)**	**0.016**	**–**	**–**	**–**
eGFR + POX	111	39	555	1.06 (0.65–1.82)	0.80	**–**	**–**	**–**	1066	**0.04 (0.01–0.15)**	<**0.001**
eGFR + UOX	150	55	–	**–**	**–**	1091	0.66 (0.38–1.09)	0.10	1250	**0.02 (0.01–0.06)**	<**0.001**
POX + UOX	106	38	550	**5.20 (2.10–14.59)**	<**0.001**	890	0.37 (0.10–1.13)	0.09	**–**	**–**	**–**
eGFR + POX + UOX	106	38	550	1.91 (0.88–4.79)	0.13	890	0.42 (0.15–1.04)	0.066	1057	**0.04 (0.01–0.14)**	<**0.001**

N = number of patients; E = number of events; eGFR = estimated glomerular filtration rate; UOX = urine oxalate; POX = plasma oxalate.

* HR can be interpreted asper doubling of the biomarker value.

Estimates in bold denote statistical significance at the 0.05 level.

Results using multiple imputation for missing data led to similar qualitative conclusions, though estimated hazard ratios were attenuated towards the null hypothesis (Supplemental Table S2). In the full multivariate joint model including POX, UOX, and eGFR, UOX was inverted compared to univariate analyses, though results were not statistically significant (HR = 0.62 [95% CI, 0.25–1.52]).

After taking fixed effects into account, UOX was positively correlated with POX (correlation of random effects for UOX vs POX: 0.59) and negatively correlated with eGFR (correlation of random effects: −0.38), while POX was negatively correlated with eGFR (correlation of random effects: −0.76). Positive correlation of two random effects suggest that patients that tend to have higher values of one biomarker also tend to have higher values of the second biomarker, while negative correlations suggest that patients that tend to have higher values of one biomarker tend to have lower values of the second biomarker (or vice versa).

### Association between POX, UOX, eGFR, and Risk of Kidney Failure at or Below CKD Stage 3a

In a sensitivity analysis, models were refit using time-dependent covariates and joint modeling after sub-setting to follow-up of patients starting at eGFR < 60 ml/min/1.73 m^2^ (CKD stage 3a) (Supplemental Tables S3 and S4). Results were similar to the main analysis in time-dependent Cox models. However, using joint modeling, POX was not statistically significant after adjusting for UOX and eGFR, while UOX was significantly negatively associated with risk of kidney failure after adjustment for both POX and eGFR. Among patients with already reduced kidney function (eGFR < 60 ml/min/1.73 m^2^), a doubling of UOX was associated with a 69% reduction in the hazard for kidney failure (HR 0.31 [95% CI 0.10–0.89]).

## Discussion

In this paper, multivariate joint models were employed for longitudinal and time-to-event data as an alternative and preferred approach for assessing the associations between biomarkers measured serially over time and time-to-event outcomes. Flexibility was permitted in model formulation and functional form relating the longitudinal biomarkers to the kidney failure event. We demonstrate that associations between longitudinal POX, UOX, and eGFR measures with time-to-kidney failure may vary based on the analysis approach and assumptions of those methods. In our analysis, the multivariate joint modeling approach is more flexible and better reflects biological plausibility than the standard LOCF method since the underlying biomarkers are not at a steady state value between observations. In LOCF models, there was a statistically significant association for POX such that a doubling of POX was associated with a 2.2 times higher hazard of kidney failure. However, in multivariate joint models, neither POX nor UOX demonstrated statistically significant associations with kidney failure. In a sensitivity analysis among those with reduced kidney function (lower eGFR), the contrast in results was more prominent. While both LOCF and joint models yielded numerically similar estimates for POX, in the multivariate joint model a doubling of UOX was associated with a statistically significant lower hazard for kidney failure (HR 0.31).

The biomarkers POX and UOX are both influenced by kidney function (i.e., eGFR). POX increases as kidney function is lost due to impaired urinary excretion, while UOX may ultimately decrease at low eGFR. These relationships are captured by the multivariate joint modeling approach, which (unlike the LOCF analysis) demonstrated an inversion of the relationship between UOX and kidney failure in advanced kidney disease. This observation has biological plausibility and may reflect loss of kidney reserve and reduced ability to excrete oxalate as POX increases. This highlights a unique advantage to this modeling framework due to an ability to capture an interdependency of different biomarker values. This advantage is preserved even when they are not measured at concurrent intervals or timepoints over time, as is often the case when using retrospective data from rare disease registries.

There are limitations to our data and analysis. First, this is a study among patients with a rare genetic metabolic disorder which limits sample size for analysis. We may be underpowered to detect clinically meaningful effects. This also limits our ability to model transitions to death via a multistate modeling approach. The Rare Kidney Stone Consortium Registry is a voluntary registry and data availability varies across participating sites. Biomarker assays (POX) are not available at all sites and data entry practices are not always consistent, leading to missing data. Additionally, results may reflect unmeasured confounding as we were not able to adjust for fluid intake or diet at baseline or changes in these over time. Finally, our analyses reflected a hypothesized association between POX and UOX values and kidney failure. Alternative relationships not considered here may include time-lagged POX or UOX or area under the UOX curve over time reflecting an accrued exposure. Supplemental Materials provides a brief description of alternative software for implementing joint models and extensions that could include these alternative relationships.

Our results may have implications for interpreting POX and UOX in relation to kidney failure risk in PH1. Results from the joint modeling approach suggest that as eGFR declines, POX may become a more sensitive indicator of oxalate burden and kidney failure risk, while the decline in eGFR and renal oxalate elimination may influence UOX and reduce its predictive value (Table 3). These trends were not as evident using the LOCF approach (Table 2). These results identified by the joint modeling approach may also have value when interpreting the prognostic value of these laboratory measures in an individual PH1 patient. While LOCF is preferred to naïve methods that do not capture changes in biomarkers over time, we encourage readers to consider broader alternatives.

## Supplementary material

For supplementary material accompanying this paper visit https://doi.org/10.1017/cts.2022.465. click here to view supplementary material

## Data Sharing

Data used in this research are part of an ongoing and active registry and are not available for public sharing or archive; requests for data may be made to Dr. John C. Lieske.
