# Supplementary material for: Application of multivariate joint modeling of longitudinal biomarkers and time-to-event data to a rare kidney stone cohort
Source: J Clin Transl Sci. 2022 Sep 26;7(1):e8. doi: 10.1017/cts.2022.465 (PMC9879888; doi:10.1017/cts.2022.465)
Supplement: Supplementary file 1 [file S2059866122004654sup001.docx]

**Supplemental Material**

References provided in the manuscript will refer the reader to a comprehensive summary of the statistical methods. Here, we summarize in brief and provide R code for our study assessing the association between longitudinal biomarkers (POX, UOX) and time-to-kidney failure.

***Multivariate Joint Modeling Framework***

We use joint modeling to link longitudinal regression models for serially ascertained biomarkers and a survival regression model for time-to event (kidney failure) outcome. In our joint model formulation, longitudinal biomarkers are evaluated using linear mixed models (longitudinal submodel). For the kidney failure process (survival model), the risk of kidney failure is linked to the longitudinal biomarkers using a subject-specific linear predictor from the longitudinal biomarker submodel. But there exists capability to fit a wide range of joint models, including flexibility in modeling the association structure between the longitudinal biomarker and time-to-kidney failure outcome by use of transformations and various functional forms, allowing for different terms from the longitudinal sub-model to enter the linear predictor of the survival sub-model. By specifying a model for the joint distribution of the serially measured biomarker and kidney failure processes, the link between the presence of the biomarker at a certain time point and its predicted future path and its relationship to kidney failure is explicitly accounted for, allowing for more accurate estimation of their association^1^.

***Equation for longitudinal biomarker submodel***

Assuming $K$ longitudinal biomarkers (in our study,$K=3$ for POX, UOX, and eGFR), we let $y_{ki}$ denote the $n_{ki}\times1$ longitudinal response vector for the $k$^th^ outcome ($k=1,\ldots,K$) for the $i$^th^ subject, with $y_{kij}$ denoting the observed value of the $k$^th^ longitudinal outcome taken at time point $t_{kij}, j=1,\ldots{,n}_{ki}.$

The regression submodels for each serially measured biomarker were fit using linear mixed effects models linked by random effects^2^. Specifically, the conditional expectation of $y_{ki}$ given random effects $b_{ki}$ is given by:

$E\left\{ y_{ki}\left( t \right) | b_{ki} \right\}= \eta_{ki}\left( t \right)= x_{ki}^{T}{(t)\beta}_{k}+z_{ki}{(t)b}_{ki}$,

where $y_{ki}\left( t \right)$ denotes the value of the $k$^th^ longitudinal outcome for the $i$^th^ subject at time $t$ and $x_{ki}\left( t \right)$ and $z_{ki}\left( t \right)$ denote fixed and random terms for fixed-effects $\beta_{k}$ and random intercepts $b_{ki}$, respectively^2^. We assume random effects $b_{i}={(b_{1i},b_{2i},\ldots,b_{Ki})}^{T}$ follow a multivariate normal distribution with mean zero and variance-covariance matrix $D$^2^.

***Equation for survival submodel***

For the time to kidney failure event process, we assume the hazard of event depends on a function of the subject-specific linear predictors $\eta_{ki}\left( t \right)$:

$$h_{i}\left( t | \eta_{i}\left( t \right),w_{i}\left( t \right) \right)= h_{0}\left( t \right)\exp\left[ \gamma^{T}w_{i}\left( t \right)+ \sum_{k=1}^{K} \alpha_{k}\eta_{ki}\left( t \right) \right],$$

where $\eta_{ki}\left( t \right)$ denotes the subject-specific estimate of the biomarker at time $t$, $h_{0}\left( t \right)$ denotes the baseline hazard function, $w_{i}\left( t \right)$ is fixed effects for the survival model for the $i$th subject. More flexible formulations have been described elsewhere, but this relationship fit our data and hypotheses well.

***Sample R code to implement univariate joint modeling using the JMbayes package***

The joint modeling function requires two separate datasets as inputs:

1. the dataset used to fit the survival sub-model, which is formatted as 1 row per subject and includes the patient’s id (“id”), event status (“event”), time to event variable (“timeeventyrs”), and other covariates of interest – here age at baseline (“agebl”) and patient sex (“sex”). This dataset is referred to as pox_surv in the sample code, and hypothetical data are shown for the first 6 subjects below:

| **id** | **agebl** | **sex** | **event** | **timeeventyrs** |
| --- | --- | --- | --- | --- |
| 1 | 9 | M | 0 | 0.5 |
| 2 | 6 | M | 0 | 11 |
| 3 | 20 | F | 0 | 5.7 |
| 4 | 8 | M | 1 | 2.5 |
| 5 | 15 | F | 0 | 5.2 |
| 6 | 7 | M | 1 | 5.8 |
| … | … | … | … | … |

1. the dataset used to fit the longitudinal sub-model, which is formatted as multiple observations per subject and includes the patient’s id (“id”), the biomarker of interest (“logpox”), the time since baseline each biomarker measure was obtained (“timeyrs”), and other covariates of interest (“agebl” and “sex”). This dataset is referred to as pox_long in the sample code, and hypothetical data are shown for the first 3 subjects below:

| **id** | **agebl** | **sex** | **timeyrs** | **logpox** |
| --- | --- | --- | --- | --- |
| 1 | 9 | M | 0 | 1.31 |
| 2 | 6 | M | 0 | 0.74 |
| 2 | 6 | M | 8.5 | 0.83 |
| 2 | 6 | M | 9 | 0.79 |
| 2 | 6 | M | 10 | 1.03 |
| 3 | 20 | F | 0 | 0.99 |
| 3 | 20 | F | 1.4 | 1.10 |
| … | … | … | … | … |

We use the JMbayes package joint model fitting function jointModelBayes() ^1^. First, submodels are fitted and initialized, then combined when the joint model is fitted. Required arguments for the survival sub-model include a survival object fit using the function coxph() of package survival ^3^ :

# Survival sub-model with main effects for age and sex, x=TRUE so design matrix included in resulting model object

coxFit.pox <- coxph(Surv(timeeventyrs, event) ~ agebl + sex , data = pox_surv, x = TRUE)

The required arguments for the longitudinal sub-model include a mixed effects object fit using either the function lme() of package nlme ^4^ or from function glmmPQL() from package MASS ^5^ :

# Longitudinal sub-model with ns, df=2 time effect, main # effects for age and sex, random intercept, outcome log(POX)

lmeFit.pox <- lme(logpox ~ agebl + sex+ ns(timeyrs, df=2), data = pox_long, random = ~ 1 | id)

Another required argument for the jointModelBayes() function is timeVar, a character string which identifies the time variable used in the mixed model:

# Joint Model with POX on the log scale

jointFit.pox <- jointModelBayes(lmeFit.pox, coxFit.pox, timeVar = "timeyrs”)

Additionally, transformations to the predictors used in the joint model can also be applied using the transFun function (did not improve fit in our report; results not shown):

# Alternative option to fit the Joint model on the biomarker’s # original scale

tf1 <- function (x, data) { "exp" = exp(x) } jointFit.pox.exp <- update(jointFit.pox, transFun = tf1)

***Sample R code for multivariate joint modeling using the JMbayes package***

The input dataset used to fit the longitudinal sub-models is similar to that used for univariate joint modeling, with the addition of the other biomarkers of interest included. The package allows for missing values to be present in the dataset for situations in which the biomarkers are obtained at different time points (see hypothetical dataset below):

To differentiate from the prior ‘long’ dataset, we give this version of the dataset the name pox_long.both.

| **id** | **agebl** | **sex** | **timeyrs** | **logpox** | **loguox** | **logegfr** |
| --- | --- | --- | --- | --- | --- | --- |
| 1 | 9 | M | 0 | 1.31 | 0.34 | 3.8 |
| 2 | 6 | M | 0 | 0.74 | . | 3.9 |
| 2 | 6 | M | 8.5 | 0.83 | 0.01 | . |
| 2 | 6 | M | 9 | 0.79 | . | 4.2 |
| 2 | 6 | M | 10 | 1.03 | . | 4.1 |
| 3 | 20 | F | 0 | 0.99 | . | 4.4 |
| 3 | 20 | F | 1.4 | 1.10 | 0.10 | . |
| … | … | … | … | … | … | … |

The input dataset for the survival sub-model for multivariate joint modeling uses the same formatting as for univariate joint modelling. A Cox model is still fit using the function coxph(),using the same survival dataset as provided above and where the model argument is set to TRUE so the model frame is returned:

# Cox model with main effects for age and sex

coxFit.poxuox <- coxph(Surv(timeeventyrs, event) ~ agebl + sex , data = pox_surv, model = TRUE)

The mixed models are now fit using the function mvlgmer()^2^ instead of the function lme(). This function follows the syntax of lmer() from the package lme4^6^.

# Longitudinal models for POX, UOX and eGFR, main effects for age and sex, random intercept, ns df=2 time effect for POX and eGFR, ns df=5 time effect for UOX, outcomes are log(POX), log(UOX) and log(eGFR)

MixedModelFit.poxuoxegfr <- mvglmer(list(loguox ~ agebl + sex + ns(timeyrs, df=5) + ( 1 | id), logpox ~ agebl + sex + ns(timeyrs, df=2) + (1 | id), logegfr ~ agebl + sex + ns(timeyrs, df=2) + (1 | id)), data = pox_long.both, families = list(gaussian, gaussian, gaussian))

The function used to fit multivariate joint models, mvJointModelBayes()^2^, has a similar syntax as the jointModelBayes() function^7^ :

# Multivariate joint model with POX, UOX and eGFR on log scale

JMFit.poxuoxegfr <- mvJointModelBayes(MixedModelFit.poxuoxegfr, coxFit.poxuox, timeVar = "timeyrs")

Transformations can also be applied to multivariate longitudinal models using slightly different syntax from the univariate models:

# Multivariate Joint model with POX and UOX on original scale

tf <- c("loguox_value" = "exp", "logpox_value" = "exp", “logegfr_value”=”exp”)

JMFit.poxuoxegfr2<-update(JMFit.poxuoxegfr, transFuns = tf)

***Multiple Imputation***

Oxalate biomarkers (POX, UOX) were not ascertained on every patient whether at baseline or during follow up. Historically and even today, POX assays are not readily available at labs nationwide or worldwide which limits collection among patients registered in those areas. Further, even when assays are performed, data entry is not always complete for all participating sites. Our primary analysis used a complete case analysis, assuming missing biomarker data was missing completely at random. As a sensitivity analysis, we also use multiple imputation assuming data are missing at random associated with other observed characteristics. Missing baseline (at PH diagnosis) values of UOX and POX biomarkers were imputed for those patients without any assessment of the respective biomarker, thus allowing inclusion in both last observation carried forward (LOCF) time-dependent Cox regression and in both univariate and multivariate joint models. Data were imputed using the chained equations approach with predictive mean matching based on age, sex, UOX, POX, eGFR, an indicator for kidney failure during follow up, and the logarithm of total follow up time. Twenty imputed datasets were created, analyses performed on each, and results combined across imputations so that the variance of the estimate reflects both between and within imputation variance.

***Other Software for Joint Modeling and Extensions***

We used an implementation in R statistical software and the JMbayes package for this analysis, with existing software suiting our hypotheses well. Other R packages implementing joint models include JM, JMbayes2, joineRML, and rstanarm ^8-11^, or users may also write customized programs suitable for their research question. Alternatives also exist in other major statistical software, including Stata stjm command^12,13^, Stata merlin command^14^, SAS JMFit Macro^15^, and SAS JM macro^16^, making the methodology accessible to a wide range of researchers.

In addition to the basic joint model functionality of the JMBayes package, which models continuous longitudinal biomarkers under an assumption that residuals are normally-distributed, other types of longitudinal responses such as skewed continuous outcomes and dichotomous outcomes can also be fit. The association structures between subjects’ longitudinal profiles and risk of an event can also be modified using a wide range of methods. For example, certain types of disease trajectories could exist in which the risk of event depends on the slope of the biomarker or the area under the longitudinal profile of the biomarker measure at a given time. Interactions among biomarker and time-independent variables (such as age or sex) in the survival model are also available. Flexibility in the survival submodel has been extended to multistate models and competing risks, recurrent events data, and interval-censored data. These models have also been implemented in a prediction framework, where predictions for the survival endpoint are dynamically updated over time based on accruing longitudinal biomarker data^17,18^.

**Figure S1.** Flowchart of inclusion criteria

**N=572** RKSC patients diagnosed with Primary Hyperoxaluria Type 1

-348 patients excluded due to:

-39 not Mayo Clinic patients

-166 PH Type 2 or 3 or unknown diagnosis

-43 no follow-up available

-100 prevalent kidney failure at PH diagnosis

**N=224** Patients from Mayo Clinic free of kidney failure at PH diagnosis and with follow-up available

-58 patients excluded due to not having any eGFR values measured aged 2 years or older prior to kidney failure or last follow-up

**N=166** Patients with at least 1 eGFR measurement aged 2 years or older available prior to kidney failure or last follow-up

**Table S1.** Estimated hazard ratios for kidney failure per doubling of the biomarker (POX, UOX, eGFR [columns]) from univariable and multivariable Cox models adjusted for age and sex using last observation carried forward (LOCF) time-dependent covariates for eGFR, POX, and UOX, in analyses with multiple imputation for missing data.

|  |  |  | **POX** | | **UOX** | | **eGFR** | |
| --- | --- | --- | --- | --- | --- | --- | --- | --- |
| **Model** | **N** | **E** | **HR (95% CI)*** | **P** | **HR (95% CI)*** | **P** | **HR (95% CI)*** | **P** |
| eGFR | 166 | 60 | - | - | - | - | **0.08 (0.02-0.28)** | **0.002** |
| POX | 166 | 60 | **1.87 (1.12-3.12)** | **0.023** | - | - | - | - |
| UOX | 166 | 60 | - | - | **1.44 (1.05-1.97)** | **0.026** | - | - |
| eGFR + POX | 166 | 60 | **1.47 (1.00-2.14)** | **0.048** | - | - | **0.11 (0.03-0.41)** | **0.005** |
| eGFR + UOX | 166 | 60 | - | - | 0.99 (0.66-1.51) | 0.99 | **0.08 (0.02-0.27)** | **0.001** |
| POX + UOX | 166 | 60 | **1.83 (1.06-3.17)** | **0.034** | 1.21 (0.79-1.83) | 0.36 | - | - |
| eGFR + POX + UOX | 166 | 60 | **1.49 (1.01-2.18)** | **0.043** | 0.93 (0.58-1.48) | 0.73 | **0.11 (0.03-0.39)** | **0.004** |

N=number of patients; E=number of events; eGFR=estimated glomerular filtration rate; UOX=urine oxalate; POX=plasma oxalate;

LOCF=last observation carried forward.

*HR can be interpreted as per doubling of the biomarker value.

Estimates in bold denote statistical significance at the 0.05 level.

**Table S2.** Estimated hazard ratios for kidney failure per doubling of the biomarker (POX, UOX, eGFR [columns]) from univariate and multivariate joint models adjusted for age and sex for eGFR, POX and UOX, in analyses with multiple imputation for missing data.

|  |  |  | **POX** | | | **UOX** | | | **eGFR** | | |
| --- | --- | --- | --- | --- | --- | --- | --- | --- | --- | --- | --- |
| **Model** | **N** | **E** | **N labs** | **HR (95% CI)*** | **P** | **N labs** | **HR (95% CI)*** | **P** | **N labs** | **HR (95% CI)*** | **P** |
| eGFR | 166 | 60 | - | - | - | - | - | - | 1285 | **0.10 (0.05-0.21)** | **<0.001** |
| POX | 166 | 60 | 610 | **2.26 (1.39-3.68)** | **0.002** | - | - | - | - | - | - |
| UOX | 166 | 60 | - | - | - | 1107 | **1.65 (1.07-2.64)** | **0.024** | - | - | - |
| eGFR + POX | 166 | 60 | 610 | 1.46 (0.87-2.43) | 0.14 | - | - | - | 1285 | **0.14 (0.06-0.37)** | **<0.001** |
| eGFR + UOX | 166 | 60 | - | - | - | 1107 | 0.93 (0.51-1.68) | 0.80 | 1285 | **0.09 (0.04-0.19)** | **<0.001** |
| POX + UOX | 166 | 60 | 610 | **2.50 (1.16-5.36)** | **0.021** | 1107 | 0.74 (0.29-1.86) | 0.50 | - | - | - |
| eGFR + POX + UOX | 166 | 60 | 610 | 1.73 (0.82-3.66) | 0.14 | 1107 | 0.62 (0.25-1.52) | 0.28 | 1285 | **0.13 (0.05-0.35)** | **<0.001** |

N=number of patients; E=number of events; eGFR=estimated glomerular filtration rate; UOX=urine oxalate; POX=plasma oxalate.

*HR can be interpreted as per doubling of the biomarker value.

Estimates in bold denote statistical significance at the 0.05 level.

**Table S3.** Estimated hazard ratios per doubling of the biomarker (POX, UOX, eGFR [columns]) from univariable and multivariable Cox models adjusted for age and sex using last observation carried forward (LOCF) time-dependent covariates for eGFR, POX, and UOX, subset to patients with reduced kidney function (eGFR<60).

|  |  |  | **POX** | | **UOX** | | **eGFR** | |
| --- | --- | --- | --- | --- | --- | --- | --- | --- |
| **Model** | **N** | **E** | **HR (95% CI)*** | **P** | **HR (95% CI)*** | **P** | **HR (95% CI)*** | **P** |
| eGFR | 92 | 48 | **-** | **-** | **-** | **-** | **0.02 (0.01-0.05)** | **<0.001** |
| POX | 61 | 31 | **3.72 (2.35-5.89)** | **<0.001** | **-** | **-** | **-** | **-** |
| UOX | 75 | 40 | **-** | **-** | **1.79 (1.22-2.63)** | **0.003** | **-** | **-** |
| eGFR + POX | 61 | 31 | **1.85 (1.12-3.07)** | **0.016** | **-** | **-** | **0.02 (0.01-0.13)** | **<0.001** |
| eGFR + UOX | 75 | 40 | **-** | **-** | 1.29 (0.83-2.02) | 0.26 | **0.02 (0.01-0.06)** | **<0.001** |
| POX + UOX | 59 | 31 | **4.44 (2.59-7.61)** | **<0.001** | 1.18 (0.68-2.05) | 0.56 | **-** | **-** |
| eGFR + POX + UOX | 59 | 31 | **1.98 (1.10-3.57)** | **0.023** | 1.38 (0.73-2.62) | 0.33 | **0.02 (0.01-0.14)** | **<0.001** |

N=number of patients; E=number of events; eGFR=estimated glomerular filtration rate; UOX=urine oxalate; POX=plasma oxalate;

LOCF=last observation carried forward.

*HR can be interpreted as per doubling of the biomarker value.

Estimates in bold denote statistical significance at the 0.05 level.

**Table S4**. Estimated hazard ratios per doubling of the biomarker (POX, UOX, eGFR [columns]) from univariate and multivariate joint models adjusted for age and sex for eGFR, plasma oxalate, and urine oxalate, subset to patients with reduced kidney function (eGFR<60).

|  |  |  | **POX** | | | **UOX** | | | **eGFR** | | |
| --- | --- | --- | --- | --- | --- | --- | --- | --- | --- | --- | --- |
| **Model** | **N** | **E** | **N labs** | **HR (95% CI)*** | **P** | **N labs** | **HR (95% CI)*** | **P** | **N labs** | **HR (95% CI)*** | **P** |
| eGFR | 92 | 48 | - | - | - | - | - | - | 767 | **0.12 (0.05-0.26)** | **<0.001** |
| POX | 61 | 31 | 378 | **2.45 (1.39-5.18)** | **<0.001** | - | - | - | - | - | - |
| UOX | 75 | 40 | - | - | - | 550 | 1.42 (0.87-2.39) | 0.16 | - | - | - |
| eGFR + POX | 61 | 31 | 378 | 1.22 (0.67-2.34) | 0.54 | - | - | - | 678 | **0.08 (0.02-0.39)** | **0.006** |
| eGFR + UOX | 75 | 40 | - | - | - | 550 | **0.52 (0.28-0.93)** | **0.028** | 742 | **0.02 (0.01-0.07)** | **<0.001** |
| POX + UOX | 59 | 31 | 375 | **4.48 (1.31-16.21)** | **0.022** | 487 | 0.37 (0.09-1.48) | 0.14 | - | - | - |
| eGFR + POX + UOX | 59 | 31 | 375 | 2.34 (0.87-7.11) | 0.10 | 487 | **0.31 (0.10-0.89)** | **0.028** | 673 | **0.05 (0.01-0.19)** | **<0.001** |

N=number of patients; E=number of events; eGFR=estimated glomerular filtration rate; UOX=urine oxalate; POX=plasma oxalate.

*HR can be interpreted as per doubling of the biomarker value.

Estimates in bold denote statistical significance at the 0.05 level.

**References**

1. Rizopoulos D. The R package JMbayes for fitting joint models for longitudinal and time-to-event data using MCMC. *Journal of Statistical Software.* 2014;72(7).

2. Rizopoulos D. Multivariate joint models: vignette for JMbayes package. <http://www.drizopoulos.com/vignettes/multivariate%20joint%20models>. Published 2018. Accessed March 31, 2022.

3. Therneau T, Lumley T. Survival: survival analysis including penalised likelihood. <http://CRAN.R-project.org/package=survival>. Published 2016. Accessed March 30, 2022.

4. Pinheiro J, Bates D, DebRoy S, Sarkar D, Team RC. nlme: linear and nonlinear mixed effects models. <http://CRAN.R-project.org/package=nlme>. Published 2016. Accessed March 31, 2022.

5. Venables W, Ripley B. *Modern applied statistics with S.* 4th edition ed. New York: Springer-Verlag; 2002.

6. Bates D, Mächler M, Bolker B, Walker S. Fitting linear mixed-effects models using lme4. *Journal of Statistical Software.* 2015;67(1).

7. Eddelbuettel D, Sanderson C. RcppArmadillo: accelerating R with high-performance C++ linear algebra. *Computational Statistics and Data Analysis.* 2014;71:1054–1063.

8. Brilleman SL, Elci ER, Novik JB, Wolfe R. Bayesian survival analysis using the rstanarm R package. *arXiv.* 2020(2002.09633).

9. Rizopoulos D. JM: an R package for the joint modelling of longitudinal and time-to-event data. *Journal of Statistical Software.* 2010;35(9):1-33.

10. Hickey GL, Philipson P, Jorgensen A, Kolamunnage-Dona R. joineRML: a joint model and software package for time-to-event and multivariate longitudinal outcomes. *BMC Medical Research Methodology.* 2018;18(1):50.

11. Rizopoulos D, Papageorgiou G, Afonso P. JMbayes2: extended joint models for longitudinal and time-to-event data. <https://CRAN.R-project.org/package=JMbayes2> Published 2022. Accessed March 31, 2022.

12. Crowther MJ, Abrams KR, Lambert PC. Flexible parametric joint modelling of longitudinal and survival data. *Statistics in Medicine.* 2012;31(30):4456-4471.

13. Crowther MJ, Abrams KR, Lambert PC. Joint modeling of longitudinal and survival data. *Stata Journal.* 2013;13(1):165-184.

14. Crowther MJ. merlin—A unified modeling framework for data analysis and methods development in Stata. *Stata Journal.* 2020;29(4):763-784.

15. Zhang D, Chen M, Ibrahim J, Boye M, Shen W. JMFit: a SAS macro for joint models of longitudinal and survival data. *Journal of Statistical Software.* 2016;71(3).

16. Garcia-Hernandez A, Rizopoulos D. % JM: a SAS macro to fit jointly generalized mixed models for longitudinal data and time-to-event responses. *Journal of Statistical Software.* 2018;84(1):1-29.

17. Rizopoulos D, Molenberghs G, Lesaffre E. Dynamic predictions with time-dependent covariates in survival analysis using joint modeling and landmarking. *Biometrical Journal.* 2017;59(6):1261-1276.

18. Andrinopoulou ER, Harhay MO, Ratcliffe SJ, Rizopoulos D. Reflections on modern methods: dynamic prediction using joint models of longitudinal and time-to-event data. *International Journal of Epidemiology.* 2021.
